# The therapeutic impact of human neonatal BMSC in a right ventricular pressure overload model in mice

**DOI:** 10.1186/s13287-020-01593-y

**Published:** 2020-03-02

**Authors:** Rong Liufu, Guocheng Shi, Xiaomin He, Jingjing Lv, Wei Liu, Fang Zhu, Chen Wen, Zhongqun Zhu, Huiwen Chen

**Affiliations:** 1 Cardiovascular Intensive Care Unit, Guangdong Cardiovascular Institute, Guangdong Provincial People’s Hospital, Guangdong Academy of Medical Sciences, Guangzhou, China; 2 Department of Cardiothoracic Surgery, Congenital Heart Center, Shanghai Children’s Medical Center, Shanghai Jiaotong University School of Medicine, Dongfang Road No. 1678, Shanghai, China

**Keywords:** Bone marrow-derived mesenchymal stem cells, Right ventricular hypertrophy, Stem cell therapy, Vascular endothelial growth factor

## Abstract

**Objective:**

To determine the impact of donor age on the therapeutic effect of bone marrow-derived mesenchymal stem cells (BMSCs) in treating adverse remodeling as the result of right ventricle (RV) pressure overload.

**Methods:**

BMSCs were isolated from neonatal (< 1 month), infant (1 month to 1 year), and young children (1 year to 5 years) and were compared in their migration potential, surface marker expression, VEGF secretion, and matrix metalloprotein (MMP) 9 expression. Four-week-old male C57 mice underwent pulmonary artery banding and randomized to treatment and untreated control groups. During the surgery, BMSCs were administered to the mice by intramyocardial injection into the RV free wall. Four weeks later, RV function and tissue were analyzed by echocardiography, histology, and quantitative real-time polymerase chain reaction.

**Results:**

Human neonatal BMSCs demonstrated the greatest migration capacity and secretion of vascular endothelial growth factor but no difference in expression of surface markers. Neonate BMSCs administration resulted in increasing expression of VEGF, a significant reduction in RV wall thickness, and internal diameter in mice after PA banding. These beneficial effects were probably associated with paracrine secretion as no cardiomyocyte transdifferentiation was observed.

**Conclusions:**

Human BMSCs from different age groups have different characteristics, and the youngest BMSCs may favorably impact the application of stem cell-based therapy to alleviate adverse RV remodeling induced by pressure overload.

## Introduction

Since their identification by Friedenstein et al. [1], bone marrow-derived mesenchymal stem cells (BMSCs) have been known for their high proliferative potential, ability to differentiate into other cell lineages [2], support of hematopoietic cells, and promotion of the secretion of various cytokines including vascular endothelial growth factor (VEGF) [3]. These impressive abilities have encouraged extensive research on stem cell-based therapies for heart diseases, such as myocardial infarction, right ventricular hypertrophy (RVH), and dilated cardiomyopathy, improving the performance of the injured heart [4]. RVH is a common complication in congenital heart disease (CHD). Prolonged hypertrophy is a risk factor for the progression of cardiac dysfunction and cardiac sudden death in children with CHD, which lacks effective treatments. The safety and efficacy of autologous BMSC transplantation paves the way for the treatment of RVH [5]. To date, large animal models have shown that intramyocardial injection of stem cells is safe and feasible [6]. The encouraging effects triggered by stem cell therapy prompted clinical trials [7], which confirmed the former hypothesis.

Despite extensive researches, the therapeutic effects have varied from experiment to experiment, and the potential mechanisms related to those effects remain unclear. There are various pathways involved in the development of cardiac hypertrophy and studies on the potential molecular mechanisms have identified therapeutic targets for the prevention of hypertrophy [8, 9]. It was reported that human BMSCs probably preserve cardiac function by inducing participation of VEGF in the anti-hypertrophic pathway in vivo [10]. However, this particular paracrine secretion of BMSC tended to have an age-associated decline, therefore limiting the potential of cell-based therapy to some extent. Additionally, the composition of MSCs changes during development and aging, and each subpopulation of the dynamic system probably has a particular function [11]. The sensitive age-associated change in characteristics and regenerative function of BMSCs is probably attributed to environmental stimuli, including extracellular matrix and circulating metabolites [12]. Some researchers have reported that the administration of allogeneic BMSCs from younger donors achieved better cardiac recovery in a myocardial infarction animal model [13, 14]. However, to date, evidence of age-associated effects on BMSC-based therapy remains insufficient.

The current study was designed to test the hypothesis that human neonatal BMSCs present the highest proliferative capacity and paracrine secretion through in vitro experiments, which favorably impact their application in RVH mice.

## Methods

### Isolation and culture of human and mouse BMSCs

The study was approved by the Institutional Ethics Committee of Shanghai Children’s Medical Center. Approximately 2 mL of bone marrow was obtained from children with various CHDs who were receiving reconstructive heart surgeries. These patients were divided into three groups according to their ages: group 1 (< 1 month), group 2 (1 month to 1 year), and group 3 (1 year to 5 years). Isolation of human marrow sample was conducted in accordance with a previously reported protocol [15]. Briefly, the bone marrow was diluted in HBSS after collection in a 20-mL syringe containing 2 mL of preservative-free heparin (400 U/mL). BMSCs were isolated from the bone marrow cells by Percoll density centrifugation. The cell suspension was adjusted and seeded at 1.8 × 10^5^ nucleated cells per cm^2^. The medium was changed every 3–4 days and BMSCs were collected at passage 3 for further experiments.

### Flow cytometry

Human BMSCs were trypsinized at passage 3, washed twice with PBS, and incubated with 0.1% bovine serum albumin (BSA) in PBS for 1 h at 4 °C with antibodies against CD34, CD45, CD 44, and CD105. Analysis was performed using a FACS flow cytometer.

### Transwell assay

For the migration assay, passage 3 BMSCs were seeded into the upper chamber of a Transwell plate with a fibronectin-coated filter (8-mm pore size, Corning Life Sciences). The bottom chamber contained medium supplemented with 10% FBS. After incubation for 18 h in 37 °C, cells adherent to the upper surface of the filter were removed. The cells attached to the bottom of the membranes were fixed with methanol and stained with crystal violet.

### Wound-healing assay

Cell migration was determined by measurement of the cells moving close to an artificial wound. Cells were wounded with a 200-μL pipette tip, washed with PBS, and incubated in medium for 12 h. The movement of cells was monitored by microscopy.

### Western blotting

Human and mouse cells were washed in PBS, harvested by centrifugation, resuspended in lysis buffer, and centrifuged at 4 °C for 5 min (1500 rpm); the lysate was then collected. Total cellular protein concentrations were determined using a BCA assay kit (Beyotime Biotechnology, China). Equal amounts (20 μg) of protein were separated by sodium dodecyl sulfate polyacrylamide gel electrophoresis (SDS-PAGE) and transferred to nitrocellulose membrane for immunoblotting. Membranes were incubated with primary antibody overnight at 4 °C and subsequently with secondary antibodies for 2 h. The signal was detected using enhanced chemiluminescence (Amersham Imager 600, USA).

### Animal model

All animal experiments were approved by the Institutional Animal Care and Use Committee of Shanghai Jiaotong University. Four-week-old male C57 mice were randomly divided into the pulmonary artery banding (PAB) and sham-operated control groups [16]. Mice were anesthetized with 2% isoflurane and performed mechanical ventilation via a volume-controlled respirator (3 mL; 45 strokes/min). Thoracotomy through the left second intercostal region was performed and the thymus was removed. After the pulmonary artery (PA) was exposed, a 26-gauge needle was placed alongside it and a 10-0 silk suture was used to secure the needle and PA together. The needle was removed rapidly and the vital signs were monitored. The thoracotomy was performed and following by thymectomy in the sham-operated group.

### BMSC transplantation

Before stem cell injection, BMSCs were harvested at passage 3 with TrypLE Express (Gibco, Life Technologies, Grand Island, NY) and resuspended in PBS. Mice were anesthetized with 2% isoflurane as previously described. Ten minutes after the PAB operation, mice underwent stem cell (*n* = 5, 1 × 10^6^ BMSCs in 30 μL) or placebo (*n* = 5, PBS 30 μL) injection to the RV myocardium using insulin syringes. Weight-based dose of BMSCs was used according to the previous dose-escalation study [17].

### Echocardiography

To evaluate heart function, echocardiography was performed on PAB and sham mice 4 weeks after surgery. Transthoracic echocardiography was performed using a Vevo 2100 digital high-frequency ultrasound system (FujiFilm Visualsonics, Toronto, ON, Canada, http://www.visualsonics.com) equipped with a probe (MS250) suited for mice imaging. Mean and peak PA gradient, right ventricular internal diameter (RVID), and RV free wall thickness were measured in the two-dimensional long-axis parasternal view by M-mode.

### Histology

Mice were sacrificed at 4 weeks after PAB and cell injections. Heart samples were fixed in 4% paraformaldehyde, dehydrated, and embedded in paraffin. Sections (7 μm) were stained with hematoxylin and eosin (H&E). Images (× 1.25 and × 200) were taken from each heart, and the diameter and measurements of RV wall thickness were analyzed. Immunohistochemical staining with α smooth muscle actin (α-SMA) antibody (Boster Biological Technology, Wuhan, China), CD31 (Servicebio, GB13428), and collagen IV (abcam, ab6586) were also performed to evaluate vascular density [18]. Vascular density was evaluated by counting the number of α-SMA, CD31, and coll IV positive small vessels at a magnification of × 200 in the free wall of right ventricle. The determination of fibrosis was showed by Masson staining, which results in fibrotic (collagen-enriched) areas appearing blue and cellular elements appearing red.

### Quantitative real-time polymerase chain reaction

In brief, total RNA samples were extracted from cultured hBMSCs at passage 3 using Trizol reagent (Invitrogen). For detecting the secretion of VEGF, VEGF receptor (VEGFR) 1 and 2, protein kinase G 1 (PKG1), and MMP-9, the mRNA was extracted from RV tissue in three groups. RNA was reverse-transcribed with reverse transcriptase (Takara) following DNase I treatment at 21 °C for 10 min. The quantitative real-time PCR was performed on a ABI 7500 Fast Real-Time PCR system (Applied Biosystems, Foster City, CA). The data were analyzed by control values from glyceraldehyde-3-phosphate dehydrogenase (GAPDH).

### Statistics

All statistical analyses were performed with GraphPad Prism 7.03 software (GraphPad Software, Inc., San Diego, California). The *t* test and one-way or two-way ANOVAs were used to analyze statistical significance. Data are expressed as the means ± SEM. A *p* value < 0.05 was considered statistically significant. The number of asterisks indicates the significance level: *p < 0.05.

## Results

### Morphological appearance and immunophenotypic analysis of BMSCs

**Fig. 1:**
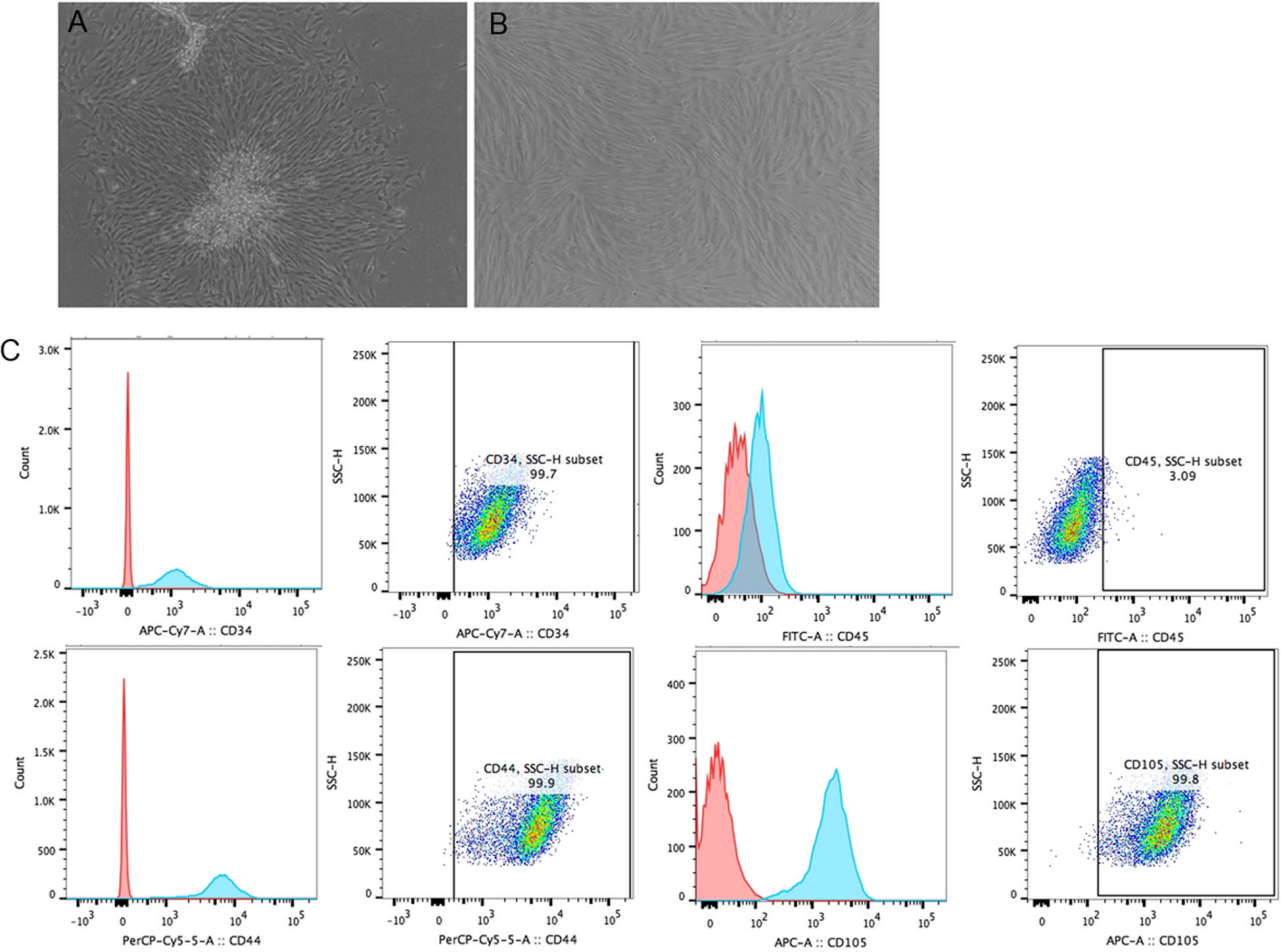
Morphology of the BMSCs. **a** Passage 0 (P0) BMSCs at × 10 magnification. **b** P3 BMSCs. **c** FACS analysis of surface markers. Typical hematologic lineage CD34, CD44, and CD105 was expressed in BMSCs, while CD45 was not

BMSCs were isolated from the bone marrow using density gradient centrifugation and cultured at 37 °C in 5% CO_2_. Cells at passage P0 demonstrated a fibroblast-like, spindle-shaped morphology (Fig. 1a) and adherence to plastic (Fig. 1b). The size of cells from different groups was not significantly different. Cell surface markers were analyzed by flow cytometry. The cell surface antigens composed of antibodies to both hematopoietic and non-hematopoietic lineage proteins for the BMSC characterization were detected. They expressed CD34, CD44, and CD105 but were negative for typical lymphocytic markers CD45 (Fig. 1c). No difference was observed in the surface markers of BMSCs between different groups.

### Migratory ability was decreased in aging BMSCs

**Fig. 2:**
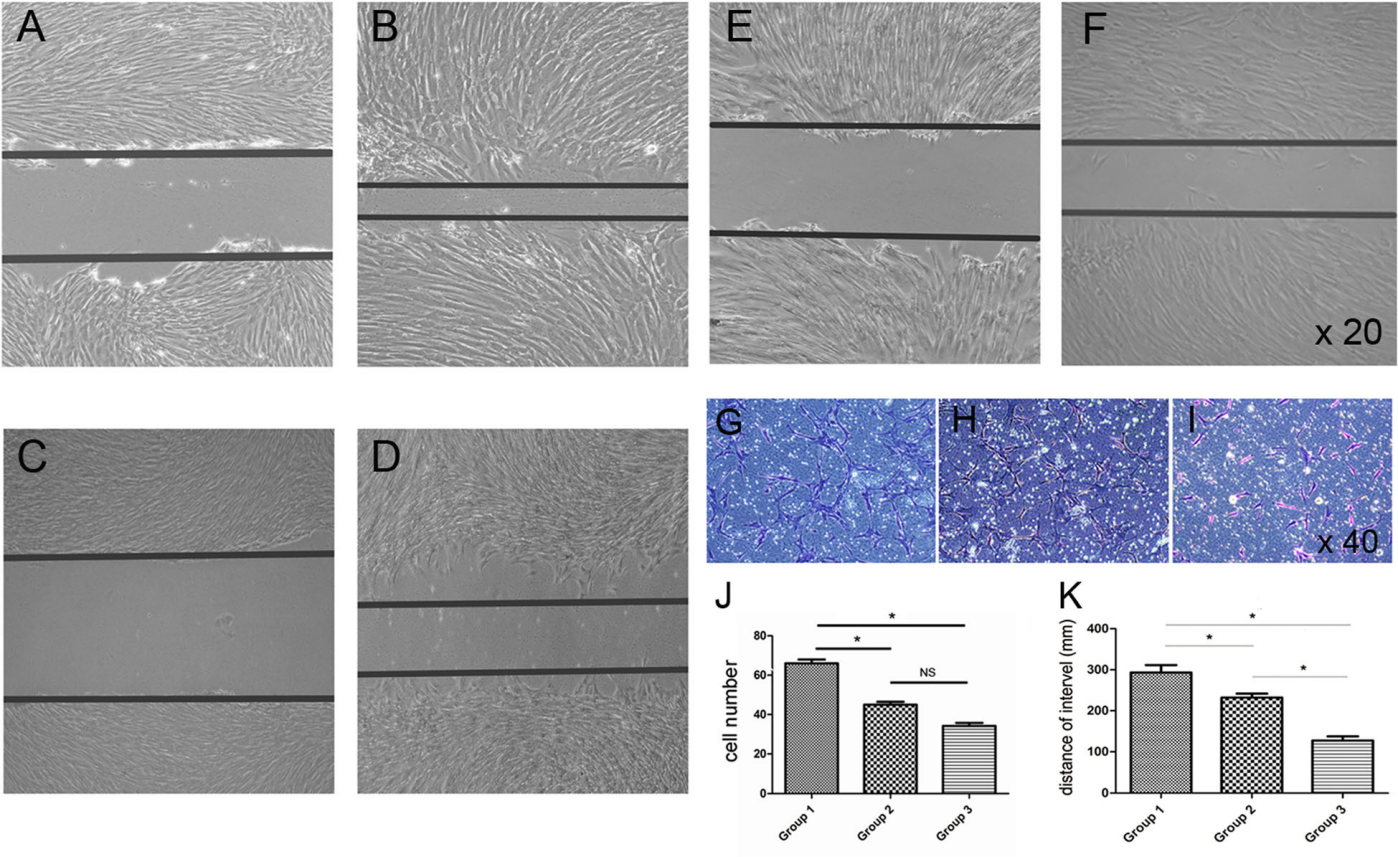
Representative images show the migration of BMSCs from group 1 (**a**, **b**), group 2 (**c**, **d**), and group 3 (**e**, **f**) in the scratch wound healing assay at 2 points. The distance of migration was measured and is shown in **k**. A Transwell assay was performed to determine the migratory ability of the BMSCs from group 1 (**g**), group 2 (**h**), and group 3 (**i**). Representative images show cell migration in the Transwell assay. The number of migratory cells was counted in six randomly chosen fields and averaged for each of the triplicate wells (**j**). *P < 0.05. NS, not significant

Next, we investigated whether BMSCs from the three groups exhibited different migration abilities by performing Transwell and scratch wound healing assays. The results show that as BMSCs aged, there was a reduction in the number of cells that cross over the filter in the Transwell assay (Fig. 2g–j), which was confirmed by the results from the scratch wound healing assay (Fig. 2a–f, k).

### BMSCs from neonate donors increased the expression of VEGF

**Fig. 3:**
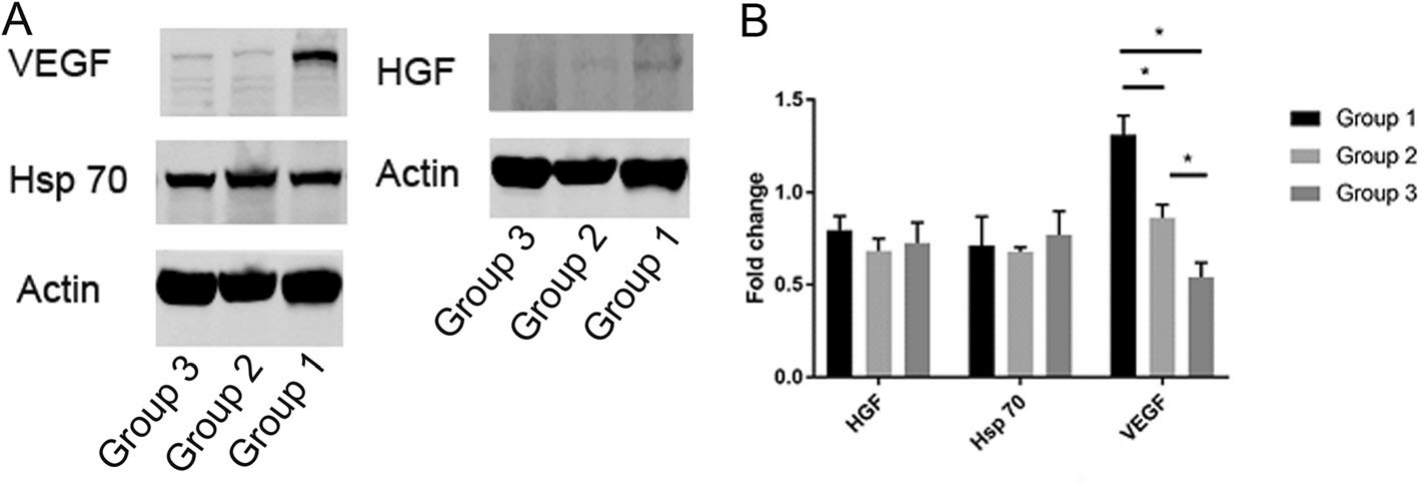
VEGF, Hsp70, and HGF expression compared among BMSCs from the three groups. **a** VEGF cytokine expression in neonate BMSCs is significantly higher than that in infant and child BMSCs, which was analyzed by western blot. **b** Quantitative real-time PCR for VEGF, HGF, and Hsp70. The results showed that the gene expression differed between the three groups. *P < 0.05

We further examined the expression of cytokines including VEGF, HGF, and Hsp70 in BMSCs from different age groups. Western blotting showed that the expression of VEGF was significantly augmented in neonate BMSCs compared to that from infant and child BMSCs, while no difference between HGF and Hsp70 expression was observed (Fig. 3a). As expected, the result was further confirmed by PCR (Fig. 3b). Younger BMSCs produced significantly higher levels of VEGF compared to the infant and child BMSCs. No difference in *HGF* and *Hsp70* gene expression or secreted protein level was observed among the three groups.

### BMSCs attenuated PAB-induced hypertrophy

**Fig. 4:**
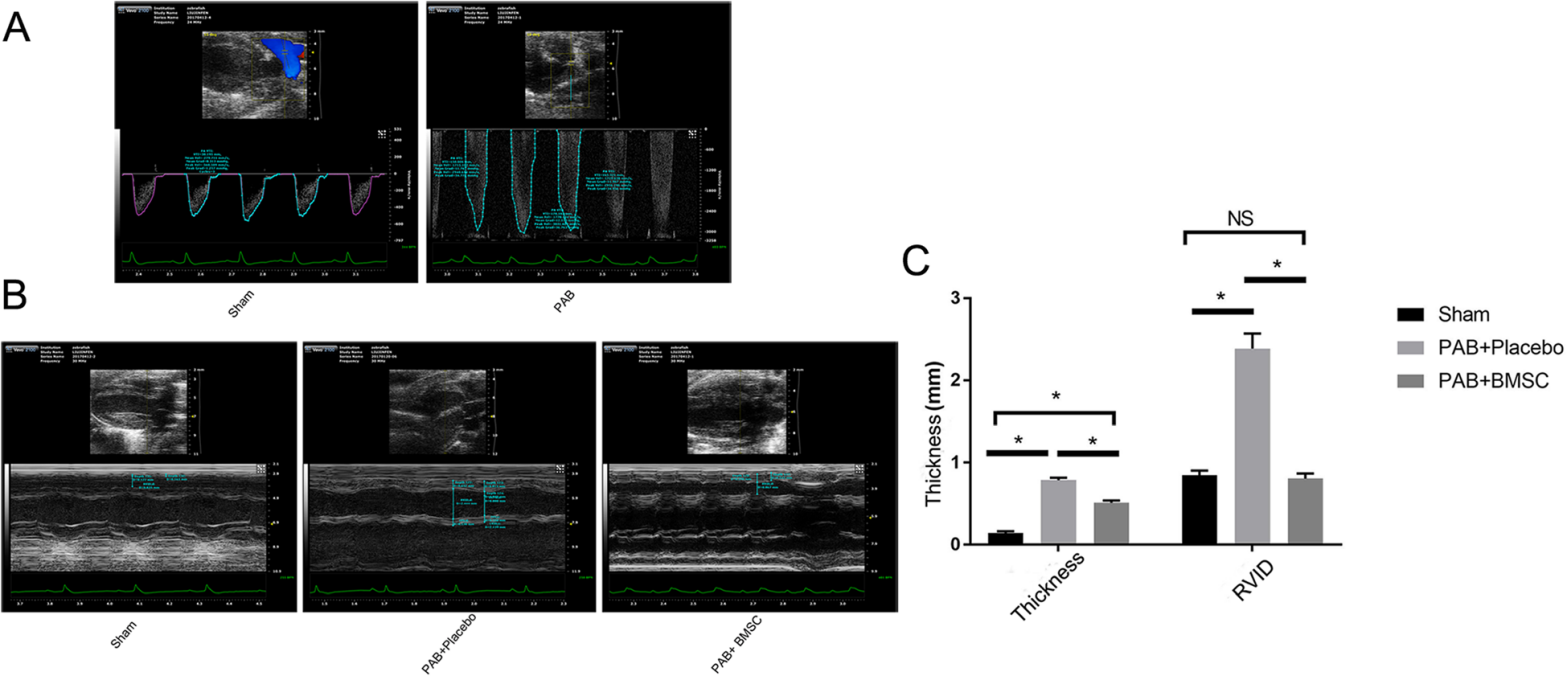
Echocardiographic assessment of RV wall thickness and RVID in mice. **a** The mean and peak PA gradient in PAB mice is higher than that in sham-operated mice. **b** Mice were divided into three groups, sham-operated, PAB with placebo treatment, and PAB with BMSC injection. Four weeks post-operation, RV wall thickness and RVID augmentation were reduced by BMSC treatment in PAB mice compared to that in the sham group. **c** Data are presented as the means ± SE; *n* = 5 in sham, *n* = 5 in PAB + placebo, and *n* = 5 in PAB + BMSCs. *P < 0.05

**Fig. 5:**
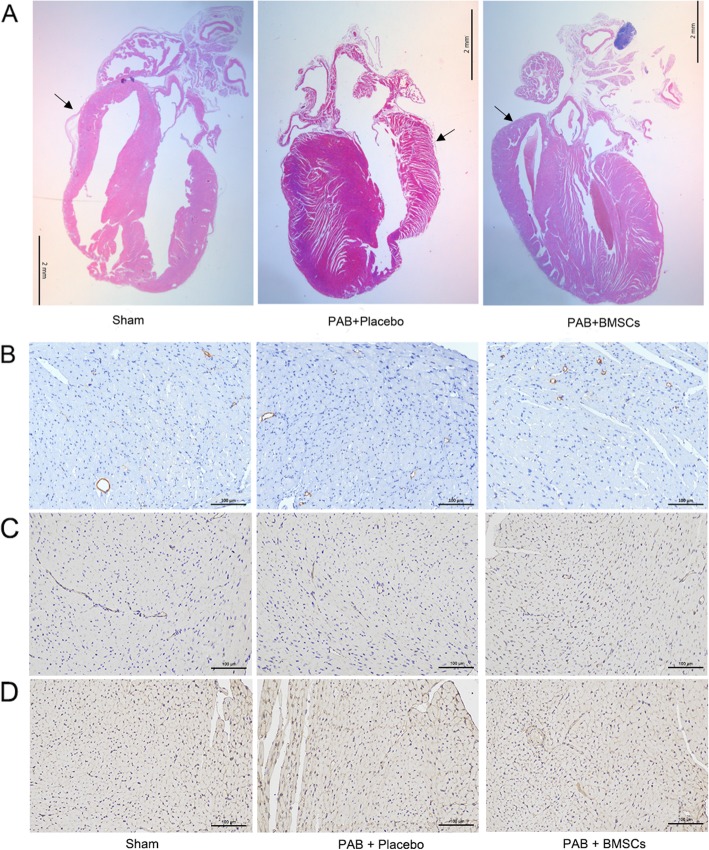
**a** Histology analysis of the three groups. The thickness of the RV wall increased after PAB operation, which can be blocked by treatment with BMSCs. The arrows indicated RV wall. **b–d** Small arteriole density was revealed in α-SMA (**b**), CD 31 (**c**), and collagen IV (**d**) by immunohistochemistry. The black arrow indicates vessels in the RV wall, which was moderately increased in the PAB with the placebo injection group. Compared to the sham-operated and PAB with placebo injection groups, small arteriole density increased slightly after BMSC injection in PA overload mice

**Fig. 6:**
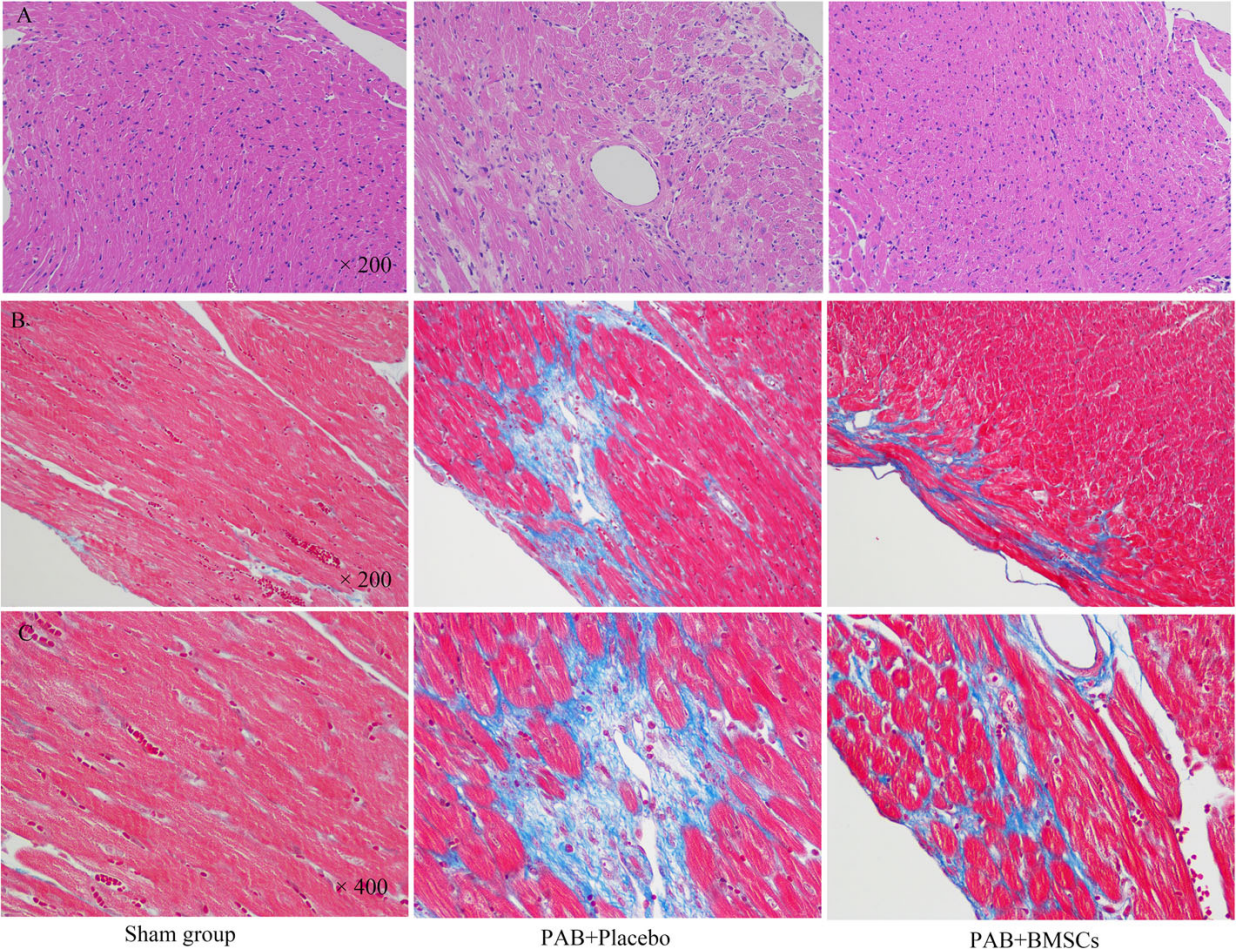
**a** H&E staining for fibrosis. **b**, **c** Masson staining for cardiac fibrosis, fibrotic (collagen-enriched) areas appearing blue, and cellular elements appearing red. The fibrosis increased after PAB surgery, while slightly increased in the hBMSC injection group

There was no operative death. Two mice suffered from pericardial effusion after the stem cell injection. Mean (11.59 ± 0.533 vs 0.35 ± 0.1 mmHg, PAB vs sham-operated; *P* < 0.001) and peak PA gradient (33.60 ± 1.09 vs 1.54 ± 0.39 mmHg; *P* < 0.001) in the PAB group were significantly increased compared to that of sham-operated animals (Fig. 4a). The PAB-induced hypertrophic effect was described as thicker RV wall and augmented RVID. This effect was attenuated by pretreatment with myocardium injection of neonate BMSCs, which also reduced the increase in RV wall thickness (0.142 ± 0.021 in sham, 0.785 ± 0.032 mm in PAB + placebo, and 0.511 ± 0.028 mm in PAB + BMSCs, *P* < 0.01) as well as RVID (0.806 ± 0.062 mm in sham, 2.389 ± 0.182 mm in PAB + placebo, and 0.847 ± 0.058 mm in PAB + BMSCs, *P* < 0.01) (Fig. 4b, c). Hearts were harvested 4 weeks after operation and treatments, and histological analysis confirmed that the hypertrophic effect can be prevented by myocardium injection of BMSCs (H&E, Fig. 5a). The arteriole density moderately increased after PAB surgery, compared to that of sham-operated mice. The increase was enhanced by the administration of BMSCs, which indicated incremental angiogenesis (Fig. 5b–d). The fibrosis increasing after PAB surgery, which was blocked in PAB and BMSCs group, confirming by high power field of H&E staining and Masson staining (Fig. 6a–c). Total collagen, which was determined by Masson staining, consistently elevated in the placebo group but remained at a normal level in neonatal hBMSC injected group (Fig. 6b, c).

### Association of VEGF signaling pathway in attenuating the hypertrophic effect

**Fig. 7:**
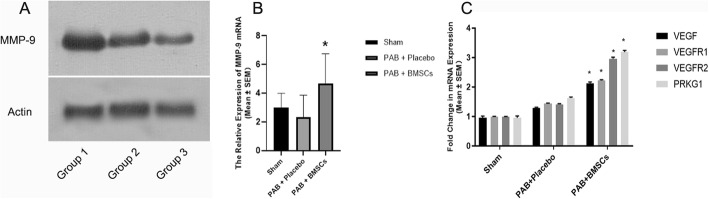
The expression of matrix metalloproteinase (MMP) 9 in vitro (**a**). Compared to infant and children group, higher migration and MMP 9 expression in neonate BMSC. Quantitative real-time PCR analysis for MMP 9, VEGF, VEGFR-1, VEGFR-2, and PKG-1 in mouse heart samples. Four weeks post-operation and treatment, heart samples were collected for PCR analysis. The results show that MMP 9 (**b**), *VEGF*, *VEGFR-1*, *VEGFR-2*, and *PKG-1* (**c**) were significantly increased in mice that received stem cell therapy after PAB, compared to those in the sham operation and PAB with the placebo treatment groups. *P < 0.05

Cardiac hypertrophy can be prevented by triggering cytokine release into the anti-hypertrophic pathway, as previously described [5, 19]. According to Zhou et al., enhanced VEGFR-1 signaling is involved in cardiomyocyte hypertrophy and the PKG-1 pathway is likely to associate with VEGFR-1. VEGF is a vital factor in formating the new vessels and re-epithelialization. Researches have reported that the positive feedback between MMP 9 and VEGF plays an important role in angiogenesis [20, 21]. It is reported that the VEGF and MMP 9 enhibit angiogenesis during the endometrium injury. By western blotting analysis, the VEGF and MMP 9 express significantly higher in the neonatal BMSC group (Fig. 7a). Thus, mRNA was extracted from RV tissue in the three groups and processed by PCR using standard protocols. The mRNA of MMP 9 were downregulation after the PAB surgery though not significantly, while the expression in the BMSC transplantation group increased (Fig. 7b). The PCR results also showed that VEGF, VEGFR-1 and 2, and PKG-1 (PRKG1) were higher in the PAB with the hBMSC injection group, compared to that in the PAB with placebo treatment or sham-operation groups (Fig. 7c).

## Discussion

In this study, we investigated the characteristics of BMSCs from different age groups and found a stronger capacity for migration and proliferation, and higher secretion of VEGF in the younger group. PAB-induced hypertrophy was prevented by neonatal BMSC transplantation in the RV wall, which was revealed by a reduction in RV thickness and RVID.

An age-related decline in expression of cytokines, BMSC proliferation, and activity seems to occur in different species, which probably limits its application to heart diseases. Upon aging, imbalanced BMSC differentiation, vascular remodeling, changes in adrenergic signaling, and inflammation influence coordinately and dynamically the stem cell and present a potential therapeutic target for age-related pathological disorders [22]. Aging characteristic compared in other studies including in vitro differentiation capacity, immunological markers, and global gene expression, which did not describe detailedly in this study. In this study, the age-related changes in BMSC characterization were dramatic despite the small age interval. Firstly, older BMSCs were associated with a significant reduction in the migration and proliferation capacity, and the dynamic expression of MMP 9 might responsible for the migration [23]. According to Wei et al., the exact mechanisms underlying BMSC migration capability are partly investigated and MMP 9 upregulation augmented the stem cells’ invasive capacity. Secondly, further experiments demonstrated that cytokine VEGF secretion was higher in neonate BMSCs than that from infants and children, which was supported by evaluation of VEGF gene expression, which is comparable to other studies [12, 24]. VEGF is a critical angiogenic factor which is associated with neovascularization, myocardial protection, and induction of anti-hypertrophic pathway signaling [5, 25]. In a previous study, after intracardiac injection of VEGF, heart function was improved and cardiomyocyte apoptosis was inhibited [9]. It was reported that the expression of many heat shock proteins was found to decline progressively with age in BMSCs from young (< 5 years old), middle-aged (8–10 years old), and old (> 12 years old) rhesus monkeys [26]. According to our data, gene expression of HGF and Hsp70 did not decrease in BMSCs isolated from older patients. The potential reason that similar results were absent in our study is because the juvenile BMSCs in this present study were isolated from a narrow range age in which the HGF and Hsp70 are not as susceptible to change as VEGF. These changes may have important consequences on myocardial healing mediated by BMSCs. The paracrine effects of BMSCs might intimately relate to its therapeutic potential to mice cardiac hypertrophy. Therefore, we suggest that BMSCs from neonates probably hold the largest potential for therapeutic effect and may present an optimal choice for stem cell treatment.

Although heart transplantation remains the surgical strategy for pediatric heart failure, the transplantation list for neonates and infants is long and the survival rate is still poor, making the therapy a rough task [27]. There are some studies demonstrated that stem cell delivery to children’s diseased hearts as an adjunct to surgical palliation provided some benefits in terms of cardiac function, somatic growth, and quality of life [7, 28]. Intramyocardial delivery of stem cells can produce higher cell retention as well as a targeted approach into the damaged myocardium [29]. Intramyocardial injection of mesenchymal stem cells in an RV pressure overload model preserved the RV function and attenuated cardiac remodeling [30]. There have also been several clinical studies applying mesenchymal stem cells on patients with hypoplastic left heart syndrome, which showed improvement of ventricular function, an increase in RV ejection fraction, and a marked decrease in brain natriuretic peptide [31–33]. However, the stem cell-based therapy remains controversial since there are disagreements regarding the therapeutic effect of stem cell injection for its inconclusive efficacy and clinical probability [34]. In our research, the hypertrophic RV model was established as previously described, significantly increasing the mean and peak PA gradient as well as the RV thickness at 4 weeks post-operation. Four weeks after placebo and neonatal BMSCs injection, echocardiography showed a significant decline in RV thickness and RVID by BMSC intervention, which was further supported by histological results. The mice that developed pericardial effusion may attribute to the rejection of human BMSCs in the murine model. Previous studies showed an extremely low retention rate in the hearts, which may cause limited outcomes [35]. Although we failed to locate the stem cells in the myocardium, a reduction in RV wall and RVID was observed and an increase in VEGF secretion was detected. The expression level of VEGF in the BMSC injection group was significantly higher than those in the control and placebo injection groups. Additionally, the enhancement of VEGF by BMSCs probably contributed to the blockade of cardiomyocyte hypertrophy. Briefly, treatment with the youngest BMSC improved cardiac function in PA overload mice, and the VEGF signaling pathway is probably associated with its beneficial effects.

Physiologic RVH improves cardiac performance in individuals; however, the persistence of the physiologic effects lays extra burden on the ventricles and readily transits to heart failure. In this research, cardiac fibrosis increased dramatically after PAB but reversed by BMSC injection. The expression of MMP 9 upregulated after BMSCs injection, which may not only affect the migration but also reconstruct the extracellular matrix [36]. The angiogenic factor interacts with VEGF since the embryo periods have a positive feedback linking to revascularization. Thus, we suggested BMSC injection caused beneficial effects in alleviating cardiac hypertrophy by paracrine cytokine and VEGF expression might be the most important part.

Researches have focused on the mechanism of hypertrophy and the anti-hypertrophic pathway, including cytokine cardiotrophin 1, growth hormone-releasing hormones, K_v_4.2 mRNA expression upregulated by basic fibroblast growth factor, and the VEGF signaling pathway [19, 37, 38]. According to a previous study, the regression of cardiomyocyte hypertrophy was related to a VEGF-dependent signaling pathway, which was also linked to PKG-1 activation [5]. Similar to other studies, we showed that the expression of VEGF, VEGFR-1, VEGFR-2, and PKG-1 was increased in the BMSCs treatment group compared to that of the control group after PAB. The anti-hypertrophic pathway was triggered by VEGF mediated by VEGFR-1, whose activation is linked to the PKG-1 signaling pathway [39]. Although still unclear, BMSCs in the myocardium of mice probably migrated to a particular area and promoted paracrine secretion, including the robust VEGF expression, leading to cardiac function preservation as well as angiogenesis. Moreover, VEGF maintained myocardial capillary density and its reductions in the vascular bed were associated with compensatory hypertrophy to heart failure [40]. Bajgelman et al. demonstrated that the application of the VEGF gene in rats revealed significantly improved cardiac function by reversal of capillary rarefaction [41]. Our results showed that the enhancement of neovascularization in BMSC administration was related to the normalization of RV wall thickness in PA overload mice. Briefly, we report that VEGF might preserve cardiac function through the anti-hypertrophic signal pathway and enhancement of angiogenesis; however, further experiments are warranted [42].

## Limitations

It is important to expand the age range of patients for the determination of different characteristics in BMSCs. However, all the bone marrow samples come from children’s hospital that underwent thoracic surgery, making the age range narrow. Further researches will involve adult patients, which covered the whole age range and thoroughly demonstrated general effect on aging BMSCs. Meanwhile, we compared the different characteristics from three age groups and found out the neonatal BMSCs might have the largest potential in cell therapy. The comparison among BMSCs from three injection groups into PAB mice has not been revealed for the following reasons. Firstly, the average weight of preoperative and postoperative (4 weeks later) mice was 9 ± 2 and 19 ± 3 g, respectively, resulting quite tiny hearts. Secondly, the heart rhythm of mice in this study ranges from 350 to 450 bpm, which makes the cells suspension or placebo injection difficult, and resulting high postoperative mortality. Larger range of cells injection was subjected to technique difficulties. Besides, we failed to locate injected stem cell in the myocardium, which resulted in insufficient evidences in location, distribution, and survival of hBMSCs after injection. The lower retention of stem cells in the myocardium and injected difficulty attributed to that. Further researches might include more appropriate animals for injection of stem cells from whole age range. Though we suggested VEGF and the signal pathway contribute to the blockage of hypertrophy, further experiments are warranted for thorough and detailed evaluation of the VEGF signal pathway.

## Conclusions

In conclusion, human BMSCs from different age groups possess different characteristics, and the youngest BMSCs have a favorable impact on the application of stem cell-based therapy using intramyocardial injections on overpressure-induced RV hypertrophy. These valuable findings encourage us to build a neonate stem cell bank that provides potential grafts for stem cell transplantation in the treatment of cardiac disorders.

## Data Availability

All data and materials were available.

## Abbreviations

BMSCs: Bone marrow-derived mesenchymal stem cells
CHD: Congenital heart disease
GAPDH: Glyceraldehyde-3-phosphate dehydrogenase
HGF: Hepatocyte growth factor
Hsp70: Heat shock protein 70
PA: Pulmonary artery
PAB: Pulmonary artery banding
PKG1: Protein kinase G 1
RV: Right ventricular
RVH: Right ventricular hypertrophy
RVID: Right ventricular internal diameter
SDS-PAGE: Sodium dodecyl sulfate polyacrylamide gel electrophoresis
VEGF: Vascular endothelial growth factor
VEGFR: VEGF receptor
α-SMA: α smooth muscle actin
